# BAM15 as a mitochondrial uncoupler: a promising therapeutic agent for diverse diseases

**DOI:** 10.3389/fendo.2023.1252141

**Published:** 2023-10-11

**Authors:** Guoji Xiong, Kexin Zhang, Yujie Ma, Yixin Song, Wenqiang Zhang, Tongbing Qi, Hongyan Qiu, Junfeng Shi, Chengxia Kan, Jingwen Zhang, Xiaodong Sun

**Affiliations:** ^1^ Department of Endocrinology and Metabolism, Affiliated Hospital of Weifang Medical University, School of Clinical Medicine, Weifang Medical University, Weifang, China; ^2^ Clinical Research Center, Affiliated Hospital of Weifang Medical University, Weifang, China; ^3^ Department of Pathophysiology, School of Basic Medical Sciences, Weifang Medical University, Weifang, China; ^4^ Department of Pathology, Affiliated Hospital of Weifang Medical University, Weifang, China

**Keywords:** subcellular organelles, energy homeostasis, mitochondrial, metabolic disease, obesity, diabetes, cardiovascular disease

## Abstract

Subcellular organelles dysfunction is implicated in various diseases, including metabolic diseases, neurodegenerative diseases, cancer, and cardiovascular diseases. BAM15, a selective mitochondrial uncoupler, has emerged as a promising therapeutic agent due to its ability to enhance mitochondrial respiration and metabolic flexibility. By disrupting the coupling between electron transport and ATP synthesis, BAM15 dissipates the proton gradient, leading to increased mitochondrial respiration and energy expenditure. This review provides a comprehensive overview of BAM15, including its mechanism of action and potential therapeutic applications in diverse disease contexts. BAM15 has shown promise in obesity by increasing energy expenditure and reducing fat accumulation. In diabetes, it improves glycemic control and reverses insulin resistance. Additionally, BAM15 has potential in non-alcoholic fatty liver disease, sepsis, and cardiovascular diseases by mitigating oxidative stress, modulating inflammatory responses, and promoting cardioprotection. The safety profile of BAM15 is encouraging, with minimal adverse effects and remarkable tolerability. However, challenges such as its high lipophilicity and the need for alternative delivery methods need to be addressed. Further research is necessary to fully understand the therapeutic potential of BAM15 and optimize its application in clinical settings.

## 1 Introduction

Mitochondria, essential subcellular organelles responsible for cellular energy production via oxidative phosphorylation, have multifaceted roles in cellular processes such as metabolism, calcium homeostasis, apoptosis, and reactive oxygen species (ROS) regulation (1). Perturbations in mitochondrial function have been implicated in the pathogenesis of diverse diseases, including metabolic disorders, neurodegenerative diseases, cancer, and cardiovascular diseases (2, 3). Mitochondrial uncouplers constitute a class of compounds that can disrupt the coupling between the electron transport chain and ATP synthesis, resulting in the dissipation of the proton gradient across the inner mitochondrial membrane. This uncoupling effect stimulates mitochondrial respiration and metabolic flexibility, thereby modulating cellular energy metabolism and potentially offering therapeutic benefits across various disease contexts (4).

A particularly promising mitochondrial uncoupler that has garnered considerable attention is (2-fluorophenyl){6-[(2-fluorophenyl)amino](1,2,5-oxadiazolo [3,4-e]pyrazin-5-yl)} amine, referred to as BAM15. BAM15 has emerged as a potent and selective mitochondrial uncoupling agent that enhances mitochondrial respiration by decoupling oxidative phosphorylation (5). Distinguished from traditional uncouplers, BAM15 exhibits distinctive features, including heightened mitochondrial targeting efficiency and reduced off-target effects, rendering it an attractive candidate for therapeutic intervention (6). These effects hold considerable therapeutic implications for various diseases, encompassing metabolic disorders characterized by impaired mitochondrial function, energy-deficient neurodegenerative diseases, and specific types of cancer reliant on mitochondrial metabolism for growth and survival (5, 7, 8).

The present review aims to provide a comprehensive overview of BAM15 as a prospective therapeutic agent for diverse diseases (**Table 1**). We will delve into BAM15’s mechanism of action, and its impact on mitochondrial function. Furthermore, we will address the safety profile and toxicity considerations associated with BAM15, highlighting the challenges as future directions for its development and optimization of BAM15 as a therapeutic agent.

**Table 1:** Therapeutic Potential of BAM15 on specific disease contexts.

Diseases	Mechanisms	Ref.
Obesity	Reduce fat Increase energy consumption Improve mitochondrial dysfunction	(5) (5, 9) (10)
Diabetes	Reduce expression of gluconeogenesis-related enzymes Reduce glucagon levels Improve insulin resistance Improve mitochondrial dysfunction	(9) (9) (11) (10)
NAFLD	Reduce oxidative stress and mitochondrial dysfunction Inhibit NLRP3 inflammatory vesicle activation Improve liver mitochondrial biogenesis	(9) (12) (13, 14)
Sepsis	Reduce mtDNA release and mtROS generation PGC-1α activation Increase the expression of TFAM Induce M1 pro-inflammatory to M2 anti-inflammatory shift	(13) (13) (13) (14)
cardiovascular disease	Induction of biphasic changes in STAT3 activity Inhibition of NLRP3 inflammatory vesicle activation	(15) (12)

## 2 Mechanism of action of BAM15

### 2.1 BAM15 uncouples oxidative phosphorylation

**Figure 1:**
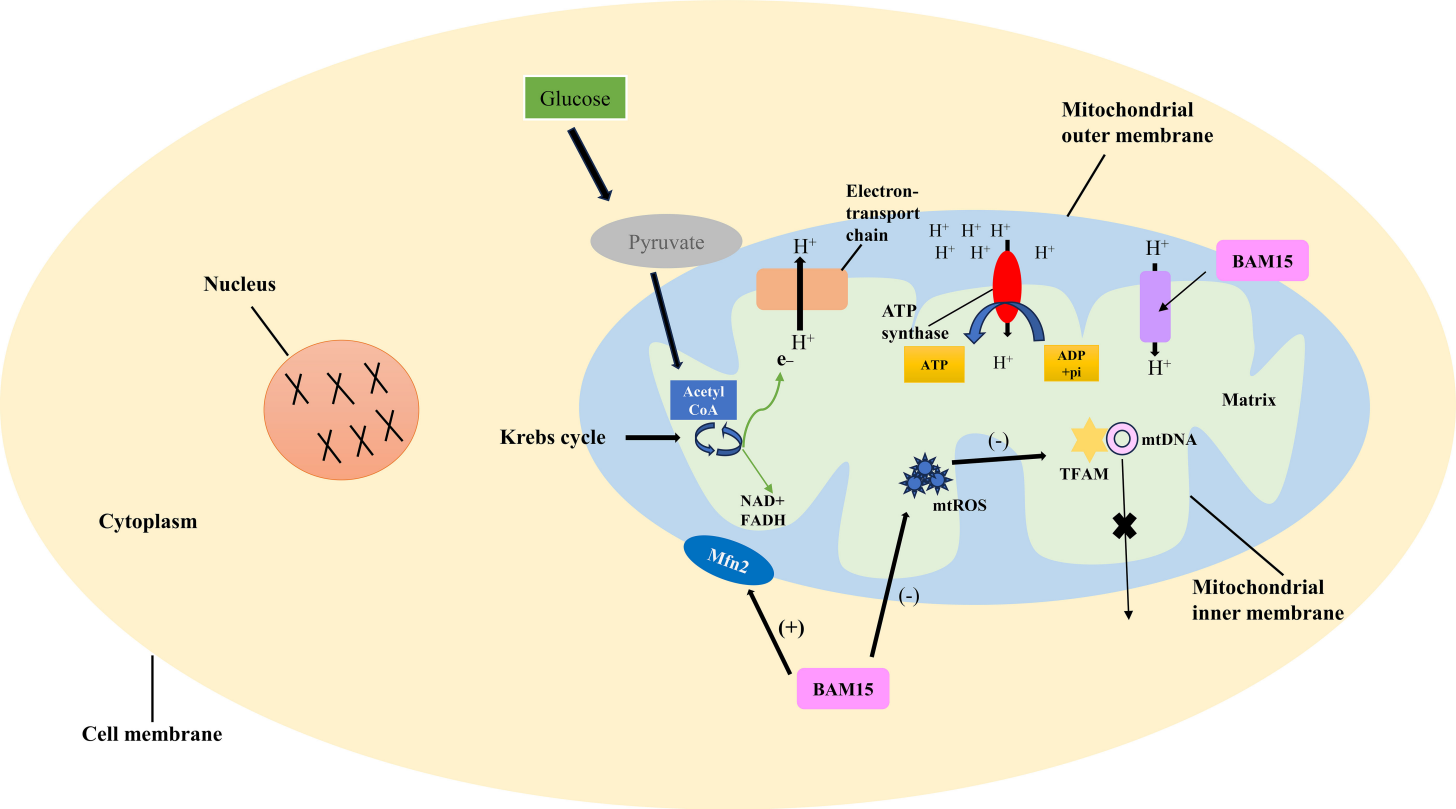
Mechanism of BAM15-induced uncoupling of oxidative phosphorylation. The link between pyruvate production, the Krebs cycle, proton release, and ATP synthesis is detailed, elucidating the BAM15-induced uncoupling process. Oxidative phosphorylation involves electron transfer through the chain, creating a proton gradient for ATP synthesis. BAM15, a synthetic mitochondrial uncoupler, disrupts this coupling. By targeting the inner mitochondrial membrane, it enhances proton permeability, dissipating the gradient and uncoupling electron transport from ATP synthesis. This boosts mitochondrial respiration and energy expenditure.

Oxidative phosphorylation involves the transfer of electrons through the electron transport chain, ultimately generating a proton gradient across the inner mitochondrial membrane. This proton gradient serves as the driving force for ATP synthesis by ATP synthase (16). The mitochondrial membrane potential is established by the electrochemical gradient of protons across the inner mitochondrial membrane, reflecting both the voltage difference (ΔΨm) and the pH gradient (ΔpH). This proton gradient is essential for facilitating ATP synthesis (17, 18) (**Figure 1**).

BAM15 is a novel mitochondrial uncoupling agent derived from a synthetic source (6). It acts by disrupting the coupling between electron transport and ATP synthesis in the mitochondria (19). By directly targeting and modulating the inner mitochondrial membrane, BAM15 enhances its permeability to protons, resulting in the dissipation of the proton gradient and uncoupling of the electron transport chain from ATP synthesis. Consequently, this uncoupling effect leads to increased mitochondrial respiration and energy expenditure. As the proton gradient dissipates, electron transport becomes less constrained, facilitating enhanced electron flow and oxygen consumption. This, in turn, promotes substrate oxidation and elevates metabolic activity to compensate for the reduced ATP production resulting from mitochondrial uncoupling, ultimately aiming to generate more ATP (9, 20).

### 2.2 BAM15 affects cellular energy metabolism and mitochondrial respiration

**Figure 2:**
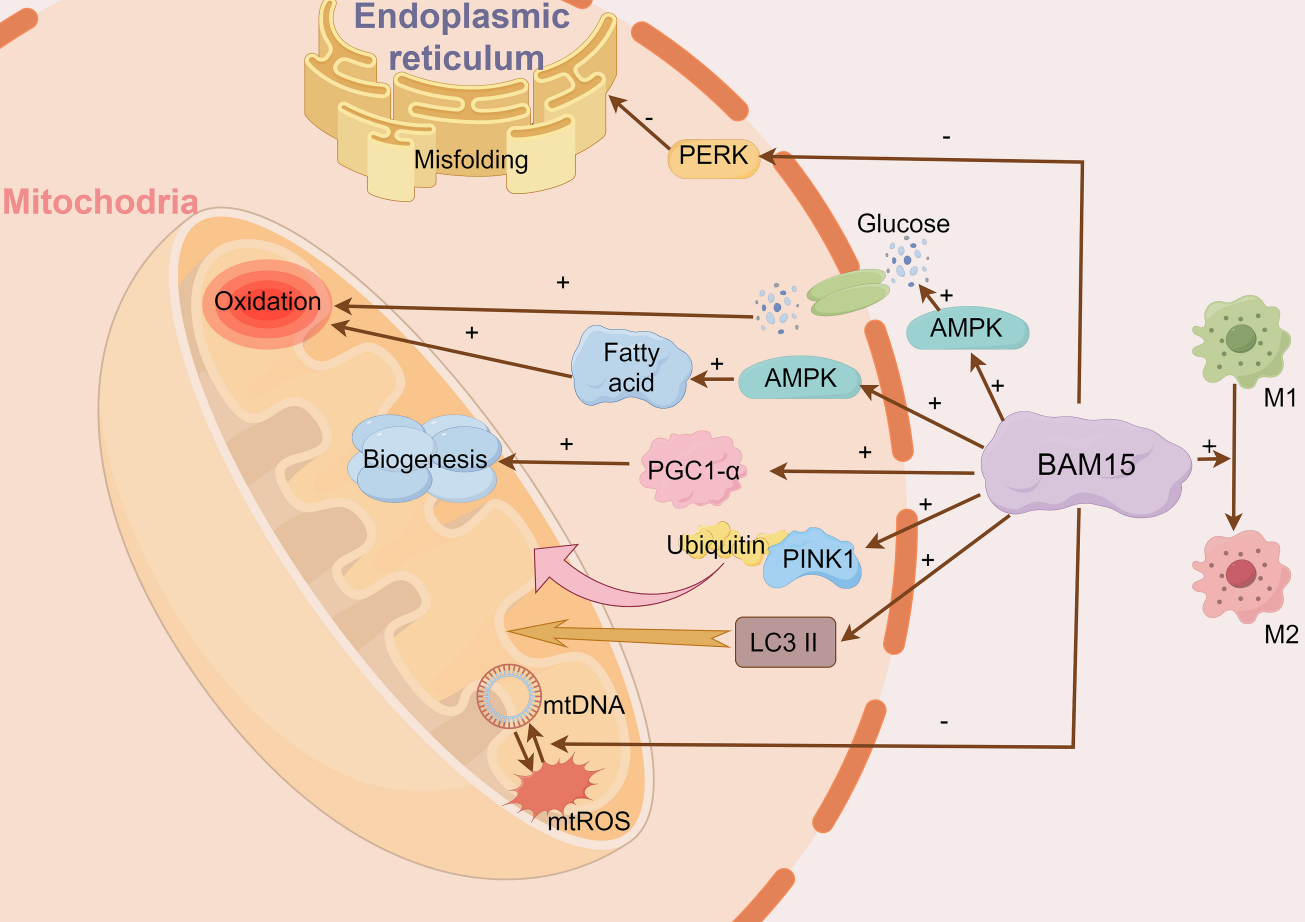
BAM15 affects cellular energy metabolism, mitigates tissue damage and suppresses inflammation. BAM15 activates pivotal signaling pathways, notably AMPK and PGC-1α. AMPK responds to BAM15-induced ATP reduction, facilitating glucose uptake and fatty acid oxidation. Concurrently, BAM15 enhances PGC-1α activity, promoting upregulation of genes related to mitochondrial biogenesis. Additionally, BAM15 mitigates tissue damage and inflammation by modulating mtDNA/mtROS release and macrophage polarization. BAM15 also initiates mitochondrial quality control processes, including PINK1-ubiquitin binding and LC3II activation.

BAM15 activates crucial signaling pathways, including AMP-activated protein kinase (AMPK) and peroxisome proliferator-activated receptor gamma coactivator 1-alpha (PGC-1α) (5, 10) (**Figure 2**). AMPK activates in response to BAM15-induced ATP depletion, promoting glucose uptake and fatty acid oxidation (21). Consequently, the activation of AMPK augments energy metabolism and mitochondrial respiration to compensate for the diminished ATP synthesis resulting from mitochondrial uncoupling (5). Moreover, BAM15-mediated uncoupling stimulates the expression and activity of PGC-1α, a key modulator of mitochondrial biogenesis and oxidative metabolism (13, 22). The heightened activity of PGC-1α triggers the upregulation of genes involved in mitochondrial respiration, mitochondrial biogenesis, and antioxidant defenses, ultimately bolstering cellular energy metabolism and mitochondrial function (22).

Furthermore, BAM15 initiates mitochondrial quality control processes, including PINK1-ubiquitin binding and LC3II activation, which result in heightened mitochondrial activity (10). This is substantiated by the observed enhancements in citrate synthase and complex II activity. BAM15 also curtails endoplasmic reticulum misfolding and alleviates endoplasmic reticulum stress through the reduction of PERK signaling (10). Additionally, BAM15 dampens apoptotic signaling pathways, as demonstrated by decreased cytochrome C release and Caspase-3/9 activation (10). In summary, BAM15 significantly impacts cellular energy metabolism and mitochondrial respiration through its intricate modulation of these crucial processes.

### 2.3 BAM15 mitigates tissue damage and suppressing inflammation

Mitochondrial DNA (mtDNA) is considered a damage-associated molecular pattern. It is released from damaged cells, and can serve as a biomarker for tissue injury. Furthermore, the release of mtDNA initiates a positive feedback process that results in mitochondrial reactive oxygen species (ROS) production, which causes the release of additional mtDNA resulting in more tissue damage. BAM15 plays a role in inhibiting this process, thereby reducing mtDNA release and excessive mtROS production, effectively mitigating tissue damage (13) (**Figure 2**).

Macrophage activation encompasses two phenotypes known as M1 and M2 polarization. M1 polarization triggers a pro-inflammatory response, while M2 polarization induces an anti-inflammatory response (23). Glycolytic activity is crucial for M1 polarization. BAM15 can facilitate the conversion of M1 pro-inflammatory macrophages to M2 anti-inflammatory macrophages by inhibiting glycolytic activity, thereby promoting an anti-inflammatory response. Additionally, BAM15 can downregulate genes associated with M1 polarization while upregulating genes associated with M2 polarization, effectively counteracting the inflammatory response (14).

Overall, BAM15 functions as a mitochondrial uncoupler by disrupting the coupling between electron transport and ATP synthesis within mitochondria. By dissipating the proton gradient, BAM15 enhances mitochondrial respiration, metabolic activity, and energy expenditure, offering potential therapeutic benefits in various disease contexts characterized by mitochondrial dysfunction (11).

## 3 Therapeutic potential of BAM15 on specific disease contexts

### 3.1 BAM15 and obesity

Obesity, characterized by excessive body fat accumulation, poses significant health risks such as heart disease, specific cancers, and musculoskeletal disorders (24–26). While traditional measures to combat obesity, including exercise, caloric restriction, and bariatric surgery, have demonstrated efficacy, their limitations in terms of patient adherence, cost, risks, and adverse effects necessitate alternative approaches (27–34). BAM15 offers a compelling alternative by effectively increasing energy expenditure and reducing fat accumulation (5). Extensive research has focused on the potential of BAM15 to improve mitochondrial function, facilitate weight loss, and promote metabolic improvements (5, 10, 35).

Mitochondria form a dynamic network, and the balance between fusion and fission determines the morphology of this network. Sarcopenic obesity often exhibits low levels of mitochondrial fusion protein 2 (Mfn2), leading to a fragmented mitochondrial network. BAM15 addresses this issue by enhancing Mfn2 expression, promoting mitochondrial network contacts, and alleviating endoplasmic reticulum stress and apoptosis (10). Furthermore, BAM15 may reduce the expression of mitochondrial fission proteins (fission 1, Mid49, and Mid51), thereby decreasing mitochondrial division in patients with obesity (10, 36). Besides its impact on mitochondrial dynamics, sarcopenic obesity can impair mitochondrial autophagy, which selectively clears damaged mitochondria through various pathways, including the phosphatase and tensin homolog (PTEN)-induced kinase 1 (PINK1)-mediated ubiquitin-dependent pathway (37). BAM15 enhances PINK1-mediated mitochondrial autophagy and improves mitochondrial biogenesis by increasing AMPK-induced activation of PGC-1α (10).

In obesity, the expansion of white adipose tissue leads to the release of excessive free fatty acids (FFA) into non-adipose organ cells, resulting in lipotoxicity and increased ROS production (38). BAM15-induced uncoupling reduces ATP production, leading to AMPK activation, which in turn enhances fatty acid oxidation, effectively mitigating ROS production by curbing electron leakage from the respiratory chain (9, 39). *In vitro* studies have shown that BAM15 improves insulin action, and enhances glucose uptake by sustained activation of AMPK (5). It also reduces the expression of adipogenesis-regulating genes, specifically sterol regulatory element binding transcription factor 1 (Srebf1) and carbohydrate response element-binding protein (CHREPB), as well as their downstream targets, stearoyl-CoA desaturase-1 (Scd1) and fatty acid synthase (Fasn). This reduction leads to the phosphorylation of Sterol regulatory element binding protein (SREBP), a key adipogenic transcription factor, subsequently inhibiting the transcriptional regulation and activation of its target adipogenic genes (5).

Furthermore, BAM15 is significantly taken up into the portal system, resulting in increased lipolysis and depletion of hepatic lipids, ultimately reducing adiposity and enhancing energy expenditure (5). As an energy expenditure agonist, BAM15 increases oxygen consumption (VO_2_), primarily through fat oxidation rather than an increase in physical activity (9). Importantly, BAM15’s effect remains independent of changes in food intake or body temperature, distinguishing it from lifestyle changes or pharmacological interventions that regulate body weight by suppressing food intake and/or appetite, potentially reducing lean body mass (5).

In conclusion, BAM15 presents a promising therapeutic approach for obesity, with the potential to increase energy expenditure and reduce fat accumulation without altering food intake or body temperature. Its effects on cellular energy metabolism and mitochondrial respiration provide valuable insights into its potential therapeutic applications. Further research is required to elucidate the specific mechanisms and optimize the use of BAM15 as a therapeutic agent in different disease contexts.

### 3.2 BAM15 and diabetes

Diabetes is a chronic metabolic disease, marked by chronic hyperglycemia. The prevalence of diabetes has increased significantly in the last decades (40, 41). Type 2 diabetes (T2D) is the most common type of diabetes and its pathogenesis involves insulin resistance and pancreatic β-cells dysfunction (42, 43). Emerging research suggests that the hyperresponsiveness of pancreatic β-cells to unfavorable environmental factors resulting in hyperinsulinemia may act as a precursor for insulin resistance development, subsequent β-cell failure, and ultimately the onset of T2D (43, 44).

Given the shared pathophysiological pathways of obesity and T2D, BAM15 displays potential as a therapeutic option for T2D. Much of BAM15’s mechanism of action aligns with its effects on obesity. Specifically, BAM15 exhibits potent insulin-sensitizing effects, leading to reversal of insulin resistance across various tissues in db/db mice or diet-induced obese mice (5, 9, 11). High-dose BAM15 effectively ameliorates metabolic issues in db/db mice, reducing liver triglycerides, improving blood glucose control, while leaving lean muscle mass and food intake unaffected (9). Additionally, BAM15 has the capacity to improve glucagon secretion and decrease glucagon concentrations. Glucagon, as a counter-regulatory hormone to insulin, promotes hepatic glycogenolysis and gluconeogenesis, thereby elevating blood glucose levels. BAM15 effectively lowers glucagon secretion and hepatic glucose output, alleviating hyperglycemia, all while preserving pancreatic alpha cell mass. This effect is attributed to the downregulation of critical enzymes in late hepatic gluconeogenesis, specifically glucose-6-phosphatase and fructose-1,6-bisphosphatase (9). Nevertheless, there is currently no research available regarding whether BAM15 influences β-cell function or promotes insulin secretion.

### 3.3 BAM15 and NAFLD

NAFLD is a highly prevalent chronic liver disease worldwide, progressing from early steatosis to non-alcoholic steatohepatitis (NASH), advanced cirrhosis, and hepatocellular carcinoma (45). Insulin resistance plays a crucial role in the pathogenesis of NAFLD by promoting hepatic lipogenesis and adipose tissue lipolysis (46, 47).

Impaired fatty acid β-oxidation in NAFLD disrupts peroxisome and cytochrome oxidation, resulting in increased ROS production, oxidative stress, and mitochondrial damage (48). However, preclinical studies have shown that BAM15 can potentially alleviate oxidative stress and mitochondrial dysfunction in NAFLD by reducing ROS production and electron leakage in db/db mice, accompanied by reduced liver and serum triglyceride concentrations (9). Meanwhile, in isolated rat liver mitochondria, BAM15 led to a collapse in membrane potential, elevated respiration rate, and triggered Ca2+ efflux (20). Similarly, *in vivo* experiments have demonstrated that BAM15@BSA NPs, a functionalized drug-albumin nanocomposite prepared by co-assembling BAM15 with bovine serum albumin, exhibit a remarkable ability to target the liver, leading to potent anti-obesity effects (49). Importantly, it proved highly effective in mitigating hepatic steatosis and enhancing the overall therapeutic efficacy for the treatment of NAFLD (49).

Activation of NLRP3 inflammasome, triggered by danger signals such as damage-associated molecular patterns (DAMPs), further contributes to NAFLD pathogenesis by inducing a pro-inflammatory response (50). Interestingly, BAM15 can inhibit the degradation of IκBα and the nuclear translocation of NF-κB by activating AMPK, thereby inhibiting the activation of NLRP3 inflammasome (12, 51). Additionally, in the lipopolysaccharide -induced sepsis mouse model, BAM15 reduced the expression of pro-inflammatory factors (IL-6, TNF-α, IL-10, iNOS) (14).

NASH is associated with hepatocyte injury characterized by ballooning, lobular inflammation, and often fibrosis. In the NASH mouse model, BAM15 demonstrated the ability to reduce liver triglyceride levels and improve liver enzymes, inflammation, and fibrosis (52). Remarkably, these beneficial effects were achieved without affecting body temperature and food intake. Collectively, these findings underscore the therapeutic potential of BAM15 for NAFLD/NASH.

### 3.4 BAM15 and sepsis

Sepsis, a life-threatening condition, is characterized by uncontrolled host response to infection, resulting in dysregulated organ function (53). Notably, sepsis is associated with the release of mtDNA into the circulation. This release triggers a positive feedback loop that initiates mtROS production, subsequently leading to the release of additional mtDNA and contributing to kidney damage (13). However, the compound BAM15 disrupts this loop, reducing mtDNA release, lowering mtROS levels, and mitigating kidney damage (13). Furthermore, BAM15 facilitates faster electron transfer in the electron transport chain, resulting in decreased mitochondrial superoxide production, subsequent attenuation of mtROS generation, and reduced kidney damage (13).

The transcriptional coactivator PGC-1α is vital for mitochondrial biogenesis regulation. Studies indicate that BAM15 may activate AMPK in the early stages of septic acute kidney injury (AKI) and silent information regulator sirtuin 1 (SIRT1) in the later stages, thereby increasing NAD+ levels and promoting PGC-1α production. Ultimately, this limits sepsis-induced AKI by enhancing mitochondrial biogenesis. Another downstream factor of PGC-1α is mitochondrial transcription factor AMA (TFAM), which interacts with mtDNA and activates mitochondrial transcription, playing a critical role in AKI. Reduced TFAM expression is observed in patients with septic AKI, and depletion of TFAM leads to mitochondrial DNA leakage, triggering the innate immune response. BAM15 may promote TFAM expression in septic kidneys by reducing mtROS, thereby inhibiting septic AKI progression (13).

Sepsis involves immune dysfunction with crucial roles for neutrophils and macrophages (13, 23). BAM15 hampers neutrophil infiltration in the kidney, reducing renal injury and enhancing net neutrophil survival (13). In sepsis hyperinflammation, macrophages mostly exhibit M1 polarization. BAM15 decreases macrophage glycolytic activity, inhibiting M1 macrophage polarization, and reducing the inflammatory response. BAM15 also regulates macrophage polarization genes, shifting macrophages from M1 pro-inflammation to M2 anti-inflammation, aiding inflammation resolution (14).

### 3.5 BAM15 and cardiovascular disease

NLRP3 inflammasomes are multimeric protein complexes implicated in the pathogenesis of various diseases, including cardiovascular conditions (54). Notably, BAM15 specifically targets the initiation step of NLRP3 inflammasome activation (12). In macrophages, BAM15 suppresses the translocation of NF-κB into the nucleus, thereby inhibiting the expression of NLRP3 and IL-1β (12). BAM15 also increases intracellular calcium concentration ([Ca2+]i) and inhibits IkBα phosphorylation, thereby blocking NF-KB translocation to the nucleus (12).

STAT3, a critical member of the STAT family, plays a crucial role in cardiac protection and homeostasis (55). It exhibits a protective effect on the heart, safeguarding it against acute ischemic injury (56). Upon phosphorylation, STAT3 forms a homodimer and translocates to the nucleus, activating the transcription of target genes. Interestingly, BAM15 elicits biphasic effects on STAT3 activity in cardiomyocytes. Low doses of BAM15 activate STAT3 through the mitoROS/JAK/STAT3 pathway, specifically inducing Tyr705 phosphorylation, resulting in reduced injury and increased ATP production. Conversely, high doses of BAM15 hinder STAT3 activation by blocking AMPK-induced Ser727 phosphorylation, leading to decreased ATP production and cardiomyocyte injury (15). Thus, BAM15 shows promise as a therapeutic candidate for cardiovascular disease.

### 3.6 BAM15 and other diseases

Recent literature highlights the importance of mitochondria in cancer initiation and progression due to their involvement in energy production, biosynthesis, and apoptosis (57, 58). Dysfunctional mitochondria intersect with various cancer signaling pathways, making them an attractive target for cancer therapy. BAM15 has shown promise in selectively targeting tumor cells in specific cancer types that rely on mitochondrial metabolism for growth and survival (7). One such cancer is breast cancer, characterized by its heightened metabolic adaptability and diverse energy synthesis pathways (7, 59). Research by Zunica et al. sheds light on how BAM15-induced uncoupling leads to sustained depolarization of ΔΨm (mitochondrial membrane potential), thus restraining ATP-linked oxidative phosphorylation (OXPHOS) in cancer cells and tumors (7). This process increases superoxide production, enhances caspase-3/7 activity, ultimately impeding tumor progression. Moreover, BAM15 is a key player in cancer treatment by disrupting mitochondrial function in melanoma cells with mutated RAS-MAPK pathways, leading to enhanced apoptosis and potential therapeutic benefits (60). This approach holds promise as a novel therapeutic strategy for combating cancer by targeting multiple aspects of mitochondrial function alongside oncogenic MAPK pathway inhibition.

Concurrently, acute myeloid leukemia (AML), a formidable myeloid hematopoietic malignancy, presents significant therapeutic challenges (61, 62). Studies suggest that BAM15 induces apoptosis and inhibits proliferation in AML cells by influencing mitochondria, disrupting the balance of ROS, and curtailing ATP synthesis, thus alleviating AML. Notably, *in vivo* experiments underscore BAM15’s effectiveness in restraining AML growth and extending the lifespan of mice, positioning it as a potential therapeutic contender against AML (63).

Neurodegenerative disorders, such as Parkinson’s disease and Alzheimer’s disease, are intricately linked to mitochondrial dysfunction within neuronal cells (64–66). Within the realm of neurodegenerative ailments, BAM15 demonstrates its potential as a neuroprotective agent by enhancing mitochondrial respiration and energy generation (8). In summary, these findings collectively propose that BAM15 holds promise as a versatile approach to addressing neurodegenerative disorders, inhibiting cancer proliferation, and combating AML.

## 4 The differences between BAM15 and other uncouplers

Mitochondrial uncoupling agents are compounds that facilitate mitochondrial uncoupling, altering the energy conversion process within mitochondria by regulating the mitochondrial membrane potential, thereby promoting metabolism (67). Various mitochondrial uncoupling agents have been discovered, influencing biological processes and diseases (68). For example, adenine nucleotide translocase (ANT), facilitates ATP/ADP exchange across the mitochondrial membrane while also exerting some decoupling effect, although the precise mechanism isn’t fully understood (69). Classical mitochondrial uncouplers, such as carbonyl cyanide p-trifluoromethoxyphenyl hydrazone and carbonylcyanide-3-chlorophenylhydrazone, are commonly used and can traverse biofilms, enabling proton passage (70). However, their impact on the plasma membrane varies with species, cell type, and other factors (70). BAM15 sets itself apart from traditional uncoupling agents. Unlike conventional ones that often have unintended membrane-related effects, resulting in undesirable outcomes such as plasma membrane depolarization, mitochondrial inhibition, and cytotoxicity, BAM15 brings about mitochondrial depolarization without influencing plasma membrane depolarization. This unique capability enables mitochondria to attain a sustained peak respiration rate with minimal cytotoxicity. Remarkably, BAM15 substantially enhances the rate of oxygen consumption and is well-tolerated. It also exhibits greater specificity and promotes mitochondrial respiration across a broader range of doses compared to standard uncoupling agents.

## 5 Safety and toxicity considerations

BAM15, a potential therapeutic agent for various diseases, undergoes rigorous evaluation to ensure its safety and toxicity profile. Extensive studies have demonstrated that BAM15 exhibits remarkable tolerability and minimal adverse effects (5). *In vitro* investigations have revealed its lower cytotoxicity compared to other uncoupling agents, making it suitable for widespread use. Importantly, BAM15 selectively depolarizes mitochondria without affecting the plasma membrane potential, reducing off-target effects on plasma membrane depolarization (13). This unique property of BAM15 allows it to induce sustained maximal mitochondrial respiration rates with low cytotoxicity, even at higher concentrations where it mildly inhibits mitochondrial respiration (11, 20). Crucially, BAM15 promotes maximal mitochondrial respiration within a significant dose range without causing respiratory failure. Assessments of BAM15’s impact on biochemical and hematological indices have shown no discernible alterations indicative of tissue damage (11). Furthermore, investigations focused on skeletal muscle tissue have not identified any detrimental systemic, cellular, or molecular effects associated with BAM15 administration (5).

Pharmacokinetic studies have provided insights into BAM15’s distribution and elimination from the body. It primarily localizes in the liver and is efficiently cleared from tissues within four hours. Notably, BAM15 exhibits an oral bioavailability of 67% and a half-life of 1.7 hours. However, it has low water solubility, which is an important consideration for formulation and delivery (11).

Overall, the safety profile of BAM15 is encouraging, with its remarkable tolerability, lower cytotoxicity, selective depolarization of mitochondria, and lack of detrimental effects on various tissues. Its pharmacokinetic properties also support its potential therapeutic application.

## 6 Challenges and future directions

The current research on BAM15 has some limitations that need to be addressed for its potential therapeutic application. One major constraint is the high lipophilicity of BAM15, which hinders its long-term viability for *in vivo* administration via injection (5). To overcome this limitation, alternative delivery methods should be explored. Another knowledge gap exists regarding the mechanism of action of BAM15 in sepsis, as most studies have focused on its early stages. It is crucial to conduct comprehensive investigations targeting the mechanism of BAM15 action during the later phases of sepsis to gain a more thorough understanding (13).

Furthermore, the influence of gender differences in the response to BAM15 requires further exploration. It has been observed that female septic mice exhibit a higher survival rate compared to males, and this difference is primarily attributed to the influence of sex hormones (13). Moreover, a comparative study that assessed the therapeutic effects of caloric restriction versus BAM15 in obese mice only included male subjects (9). To unravel the underlying mechanisms and better understand how BAM15 is differentially expressed between genders, future studies should consider conducting gender-stratified analyses. This approach will help investigate potential gender-specific responses to BAM15 and shed light on the distinct effects it may have on male and female subjects.

## 7 Conclusion

In conclusion, BAM15 holds great promise as a prospective therapeutic agent for various diseases characterized by mitochondrial dysfunction. As a potent and selective mitochondrial uncoupler, BAM15 disrupts the coupling between electron transport and ATP synthesis in mitochondria, leading to increased mitochondrial respiration and metabolic activity. Its unique features, including heightened mitochondrial targeting efficiency and reduced off-target effects, make it an attractive candidate for therapeutic intervention. In obesity, BAM15 increases energy expenditure and reduces fat accumulation, offering a potential strategy for weight loss and metabolic improvements. In diabetes, BAM15 improves glycemic control, reverses insulin resistance, and enhances glucose metabolism. In NAFLD, BAM15 mitigates oxidative stress and mitochondrial dysfunction. Moreover, BAM15 shows potential for improving sepsis and septic AKI by reducing mtDNA release, mitigating mitochondrial dysfunction, and modulating inflammatory responses. Additionally, BAM15 induces biphasic changes in STAT3 activity, making it a promising candidate for cardioprotection. The safety profile of BAM15 is encouraging, with minimal adverse effects and remarkable tolerability. Overall, BAM15 represents a promising avenue for therapeutic intervention in various diseases. However, challenges remain, such as the high lipophilicity of BAM15 and the need for alternative delivery methods. Further research and exploration of BAM15’s effects and mechanisms are warranted to fully understand its therapeutic potential and optimize its application in clinical settings.

## Author contributions

GX and KZ were responsible for conceptualization, methodology, data curation, and writing-original draft preparation. JZ and XS were responsible for conceptualization, supervision, writing-reviewing and editing; others were responsible for data curation and investigation. All authors contributed to the article and approved the submitted version.
